# Recombinant pregnancy-specific glycoprotein-1-Fc reduces functional deficit in a mouse model of permanent brain ischaemia

**DOI:** 10.1016/j.bbih.2022.100497

**Published:** 2022-08-24

**Authors:** Kyle Malone, Jennifer A. Shearer, John M. Williams, Anne C. Moore, Tom Moore, Christian Waeber

**Affiliations:** a Department of Pharmacology and Therapeutics, Western Gateway Building, University College Cork, Cork, Ireland; b School of Pharmacy, University College Cork, Cork, Ireland; c School of Biochemistry and Cell Biology, University College Cork, Cork, Ireland

**Keywords:** Stroke, Immunomodulation, Ischaemia, Regulatory T cells, Pregnancy-specific glycoprotein, PSG1, Cytokine, Neuroinflammation

## Abstract

**Background:**

The well-characterised role of the immune system in acute ischaemic stroke has prompted the search for immunomodulatory therapies. Pregnancy-specific glycoproteins (PSGs) are a group of proteins synthesised by placental trophoblasts which show immunomodulatory properties. The aim of this study was to determine whether a proposed PSG1-based therapeutic enhanced recovery in a mouse model of brain ischaemia and to explore possible immunomodulatory effects.

**Methods:**

Mice underwent permanent electrocoagulation of the left middle cerebral artery (pMCAO). They received saline (n = 20) or recombinant pregnancy-specific glycoprotein-1-alpha “fused” to the Fc domain of IgG1 (rPSG1-Fc) (100 μg) (n = 22) at 1 h post-ischaemia. At 3 and 5 days post-ischaemia, neurobehavioural recovery was assessed by the grid-walking test. At 5 days post-ischaemia, lesion size was determined by NeuN staining. Peripheral T cell populations were quantified via flow cytometry. Immunohistochemistry was used to quantify ICAM-1 expression and FoxP3+ cell infiltration in the ischaemic brain. Immunofluorescence was employed to determine microglial activation status via Iba-1 staining.

Results: rPSG1-Fc significantly enhanced performance in the grid-walking test at 3 and 5 days post-ischaemia. No effect on infarct size was observed. A significant increase in circulating CD4^+^ FoxP3+ cells and brain-infiltrating FoxP3+ cells was noted in rPSG1-Fc-treated mice. Among CD4^+^ cells, rPSG1-Fc enhanced the expression of IL-10 in spleen, blood, draining lymph nodes, and non-draining lymph nodes, while downregulating IFN-γ and IL-17 in spleen and blood. A similar cytokine expression pattern was observed in CD8^+^ cells. rPSG1-Fc reduced activated microglia in the infarct core.

**Conclusion:**

The administration of rPSG1-Fc improved functional recovery in post-ischaemic mice without impacting infarct size. Improved outcome was associated with a modulation of the cytokine-secreting phenotype of CD4^+^ and CD8^+^ T cells towards a more regulatory phenotype, as well as reduced activation of microglia. This establishes proof-of-concept of rPSG1-Fc as a potential stroke immunotherapy.

## Highlights

• rPSG1-Fc enhances functional recovery in a mouse model of permanent brain ischaemia.
• rPSG1-Fc increases circulating CD4^+^ FoxP3+ cells and brain-infiltrating FoxP3+ cells.
• rPSG1-Fc increases the expression of IL-10 among CD4^+^ cells in spleen, blood, and lymph nodes.

## 1 Introduction

Acute ischaemic stroke is a leading cause of death and long-term disability worldwide (Johnson et al., 2016). Despite the devastating impact of the disease, currently there is only one approved drug (intravenous recombinant tissue plasminogen activator) for treatment (Ganesh and Goyal, 2018). Thrombectomy is another option, but while it has prolonged the time window in which patients can benefit from reperfusion therapy (24 h in select cases), this approach is not without risk and an overall low number of patients (∼10%) remain eligible (Ganesh and Goyal, 2018). It is clear novel treatments are urgently needed. The immune response plays an important role in the pathogenesis of ischaemic stroke, with innate and adaptive immune cells implicated in both stroke risk and brain injury post-ischaemia (Malone et al., 2018). Immunomodulatory therapeutic strategies are therefore a compelling target for the management of stroke (Malone et al., 2019).

Pregnancy-specific glycoproteins (PSG) are a family of proteins expressed predominantly by placental trophoblasts (Moore and Dveksler, 2014; Moore et al., 2022). Detectable in maternal blood in all trimesters, the serum levels of PSGs reach >100 μg/ml by the end of gestation, making them the major group of secreted placenta proteins (Falcón et al., 2014). Altered PSG levels may be associated with various pregnancy disorders, and these proteins have garnered interest for their diverse immunomodulatory and anti-platelet properties (Masson et al., 1983; Silver et al., 1993). PSGs impair T cell proliferation, enhance secretion of anti-inflammatory cytokines (e.g., IL-10) by macrophages, reduce pro-inflammatory factor production, and shift T cell differentiation towards a Th2-type immunity (Motrán et al., 2002, 2003; Ha et al., 2005; Rayev et al., 2017; Bebo and Dveksler, 2005). PSGs may also be strong promoters of a subpopulation of CD25+ Foxp3+ T cells termed regulatory T cells (or “Tregs”), a cell type well-characterised as contributing to post-stroke repair (Wang et al., 2021). This is most likely due to the activation of latent TGF-β (Warren et al., 2018). Increased FoxP3 expression was observed in cells incubated with PSG1 (Zamorina et al., 2016). Likewise, mice administered PSG1 showed increased numbers of Tregs in the colonic lamina propria, a response which proved protective in a model of colitis (Blois et al., 2014). Enhanced numbers of Tregs and improved recovery was also noted in a mouse model of acute graft-versus-host disease (Jones et al., 2019). Finally, vector-based expression of PSG1 in a model of rheumatoid arthritis ameliorated clinical symptoms while increasing splenic Treg cells (Falcón et al., 2014).

Taken together, these data suggest PSG1 strongly promotes the Treg phenotype and provides marked improvement in disease states characterised by excess inflammation. The primary aim of this study was to test the hypothesis that rPSG1-Fc treatment (a) enhances neurobehavioural recovery and/or (b) decreases lesion size in the post-ischaemic mouse. The secondary objective of this study was to test the hypothesis that a beneficial role of rPSG1-Fc in experimental stroke is mediated by skewing the immune response to a suppressive phenotype, as assessed by increased FoxP3+ cell counts and IL-10/TGF-β production, and reduced microglial and endothelial activation.

## 2 Methods

### 2.1 Ethics statement

Animal experiments were carried out in accordance with the European Directive 2010/63/EU, following approval by the Animal Experimentation Ethics Committee at University College Cork and the Health Products Regulatory Authority Ireland (license number AE19130/P128). The study was conducted and is reported according to the ARRIVE guidelines (Percie du Sert et al., 2020).

### 2.2 PSG protein production

The pTT3-PSG1-Fc expression vector was used to produce rPSG1-Fc protein. Briefly, the expression vector was transiently transfected into Freestyle HEK293-F cells (Thermofisher Scientific, #R79007) using Freestyle MAX reagent (Thermofisher Scientific, #16447100). The plasmid DNA was diluted in OptiPRO Serum Free Medium (Thermofisher Scientific, #12309019) at a ratio of 1 μg DNA in 20 μl OptiPRO per ml of cells. Freestyle MAX reagent was also diluted in OptiPRO at the same ratio (1 μl Freestyle MAX reagent in 20 μl OptiPRO per ml of cells). Diluted DNA and Freestyle MAX reagent were combined, mixed gently, and incubated at room temperature (RT) for 20 min. The mixture was added to the cell suspension, cultured for 72 h and then centrifuged at 1000 rpm for 5 min at RT to separate the protein-containing medium. rPSG1-Fc protein was purified from cell culture medium using a Cytiva 5 ml HiTrap Protein G column and AKTA explorer FPLC in 20 mM sodium phosphate, pH 7.0 binding buffer, and eluted with 0.1 M glycine-HCl, pH 2.7 in 3–5 ml elution volumes. Fractions were pooled and concentrated to a volume of 1 ml using a Millipore Amicon Ultra Ultracel 10K centrifugal filter (Millipore, #UFC901096). The concentrate was then gel filtrated using a HiLoad® 16/60 Superdex® 75 pg (Sigma-Aldrich, #GE28-9893-33) column and AKTA explorer FPLC, in phosphate buffered saline (PBS) at 4°C. The resultant peak was fractionated, and the purified rPSG1-Fc protein was concentrated to a volume of 1 ml and sterile filtered (0.2 μm filter). Protein was quantified by Bradford Assay or UV Spectroscopy, checked by polyacrylamide gel electrophoresis, tested for LPS contamination (Limulus Amebocyte Lysate QCL-1000; Cambrex BioScience, Germany), aliquoted and frozen at −80°C.

### 2.3 Mice

A total of 45 6-7 week-old C57BL/6J mice (Envigo, UK) were used in this study (21 males and 24 females). Mice were acclimatised for seven days before the start of the study. Mice were group-housed in individually ventilated cages in a specific-pathogen-free facility. Mice were exposed to a 12 h light/12 h dark cycle and maintained at a temperature of 20–24°C and relative humidity of 45–65%. Mice were provided with environmental enrichment and *ad libitum* access to food and water. Group sizes were determined by an *a priori* sample size calculation which calculated the number of mice required to detect a 40% improvement in grid walking test score based on the variability typically observed in this test (alpha=0.05; power = 80%) (Diaz et al., 2021). While these studies were powered to detect an improvement in behavioural outcome, data from previous studies showed n > 3 per group would be sufficient to detect the expected effect of rPSG1-Fc on Treg frequency in spleen, while n > 6 per group would be sufficient to detect the expected change of CD3^+^ cells in brain. Pre-determined exclusion criteria included mice with uncontrollable haemorrhage, or thermal or physical damage to the cortex during surgery.

### 2.4 Ischaemia model

A permanent distal middle cerebral artery occlusion model (pMCAO) was employed, as previously described (Llovera et al., 2014). Briefly, mice were anesthetized with isoflurane (3–4% for induction; 1–2% for maintenance) in O_2_/N_2_ (30%/70%). The skin between the left ear and eye was infiltrated with 0.5% bupivacaine (0.1 ml) before being incised. The temporal muscle was retracted to expose the temporal and parietal bones. A small craniotomy was performed to expose the MCA and the distal portion, including the branches and main artery below the bifurcation. The MCA was occluded by bipolar electrocoagulation (Bovie Bantam Pro electrosurgical generator (#A952)/McPherson 88.9 mm straight forceps with a 0.5 mm tip (#A842) (Symmetry Surgical Inc, USA)). The MCA was partially cut to confirm successful occlusion and the incision was sutured. Mice were allowed to recover in a heated chamber (32°C) for 30 min before being returned to their home cage. At 1 h post-ischaemia, mice received either saline (n=20) or 100 μg rPSG1-Fc (n=22) via subcutaneous injection. A researcher not associated with the surgery prepared treatment solutions for volumes no greater than 250 μl per injection. Experimental mice were monitored daily using a scoresheet (checklist score) which graded weight loss (from 0 to 3), appearance changes (from 0 to 3), behaviour (from 0 to 3), and neurological score (0: no observable deficit, 1: forelimb flexion, 2: decreased resistance to lateral push and forelimb flexion without circling, 3: same as 2, with circling) (Schaar et al., 2010).

### 2.5 Neurological deficit evaluation

Mice underwent behavioural evaluation at 3 and 5 days post-stroke. To do so, they were placed on a wire grid (25 cm x 35 cm) with 1 cm^2^ openings and encouraged to move from one end to the other where a cardboard cylinder was situated. Videos were recorded (angled from below, Canon Legria HFR706) as mice crossed the grid to allow visualisation of steps and paw placement. The first 100 steps were assessed and the number of missed contralateral and ipsilateral steps were counted by an investigator blinded to the treatment groups. The deficit score was then calculated as the ratio of ipsilateral/contralateral steps missed.

### 2.6 Tissue collection and processing

At 5 days post-ischaemia, mice were culled by anaesthetic overdose (Euthatal, 200 mg/ml i.p.; Merial). Approximately 500 μl of blood was collected from the descending aorta and transferred into EDTA-coated tubes (Cruinn Diagnostics, #262197). Mice were then perfused transcardially with 20 ml cold PBS. The brain was removed and frozen in isopentane (−42°C). Brain sections (10 μm) were cut on a cryostat at 500 μm intervals and stored at −20°C. Cervical and inguinal lymph nodes, and spleen tissue were harvested and stored in PBS, before being mechanically dissociated using the back of a plunger from a 3 ml syringe (Fisher Scientific, #14955451) in approximately 3 ml PBS in a sterile 6-well plate. The resulting cell suspensions were passed through a 70 μm cell strainer (Cruinn Diagnostics, #2272906G) and collected in a 50 ml conical tube. Wells and strainers were washed twice with 1X Dulbecco's PBS (Sigma-Aldrich, #D8537). Spleen and blood samples were resuspended in 5 ml of 1X Red Blood Cell Lysis Buffer (eBioscience, #430054) and incubated for 5 min at RT. The lysis reaction was stopped by adding 2 ml of 1X PBS. All samples were then washed twice with 1X PBS, resuspended in PBS and counted using trypan blue (Sigma-Aldrich, #T8154) to determine total cell concentration and viability.

### 2.7 Infarct size measurement

Brain sections from each mouse (n = 20–22) were analysed by NeuN immunohistochemistry to quantify infarct and oedema volume. Slides were stained according to our previously published protocol (Diaz et al., 2021). Stained sections were then scanned at 3200 dpi on an Epson Perfection V550 scanner. Lesion size and hemispheric atrophy were measured on scanned images using ImageJ (Schneider et al., 2012) (Supplemental Fig. 1). The areas (mm^2^) of interest were measured and multiplied by the distance between sections (500 μm) to calculate volume (mm^3^).

### 2.8 Flow cytometric analysis

For the analysis of intracellular cytokines, cells (2 x 10^6^ per well) were resuspended in culture medium (RPMI (Sigma-Aldrich, #R7658) + 10% FCS (Sigma-Aldrich, #F2442)) and stimulated with Cell Stimulation Cocktail (1X) (eBioscience, #00497593) at 37°C in a CO_2_ incubator. After 4 h, stimulated and non-stimulated samples were resuspended for 5 min with 50 μl of anti-mouse CD16/CD32 (Clone 93, 1:100) (eBioscience). The respective cell suspensions were then stained for anti-mouse CD45 (PerCP-CY5.5) (30-F11, 1:100), CD3 (PE-Cy7) (145-2C11, 1:100), CD4 (FITC) (RM4-5, 1:800), CD8 (Pacific Blue) (5H10, 1:100), and CD25 (APC) (PC61.5, 1:100) (all sourced from eBioscience). A live/dead stain (1:10,000 solution) was also added to each sample (Fixable Viability Dye eFluor 780) (eBioscience, #65086514). Samples were then incubated for 30 min at 2–8°C in the dark. Post-incubation, the samples were washed, fixed, permeabilised, and stained intracellularly for 30 min at RT with either anti-mouse FoxP3 (PE) (FJK-16s, 1:100) (in accordance with the manufacturer's instructions in the Mouse Regulatory T Cell Staining Kit #1 (eBioscience, #88811140)), or a cocktail of anti-IFN-γ (eFluor610, eBioscience, #61731182) (XMG1.2, 1:100), anti-IL-10 (PerCP-Cy5.5, eBioscience, #45710182) (JES5-16E3, 1:50), and anti-IL-17 (PE, BD, #559502) (TC11-18H10, 1:100). Samples were then re-suspended in an appropriate volume of PBS. Flow cytometric analysis was performed with a LSRII flow cytometer (BD). Compensation control was set using BD CompBead Anti-Rat/Anti-Hamster Particles Set (BD, #552845). Data was analysed using Flowjo (v10) according to the following initial gating strategy: live cells (as determined by live/dead stain), lymphocytes (as determined by FSC/SSC), T lymphocytes (as determined by CD3^+^, CD4^+^ and CD8^+^), and regulatory T cells (CD4^+^ cells which co-express CD25 and FoxP3). Gates were set according to unstained samples and fluorescent minus one controls. The gating strategy for final determination of intracellular cytokine staining is outlined in Supplemental Fig. 2. Absolute cell counts for all tissues were calculated in accordance with instructions provided with the CountBright Absolute Counting Beads (Molecular Probes, #C36950). All results were reported according to the Minimum Information About a Flow Cytometry Experiment (MIFlowCyt) (Lee et al., 2008).

### 2.9 Immunohistochemistry (CD3, FoxP3, ICAM)

Endothelial activation (ICAM-1 staining) and lymphocyte infiltration (CD3, FoxP3) were examined in 10 randomly selected mice per group. Sections were fixed with ice cold acetone (−20°C) for 10 min, dried and washed with PBS. Slides were incubated in blocking solution (5% Rabbit Serum/PBS for CD3/FoxP3, 10% Rabbit Serum/PBS for ICAM-1) for 30 min. For CD3 staining, endogenous biotin was blocked according to the instructions provided with the Avidin/Biotin Blocking Kit (Vector Laboratories, #SP-2001). Sections were incubated with primary antibodies against CD3 (145-2C11, 1:500), FoxP3 (FJK-16s, 1:300), or ICAM-1 (1A29, 1:200) (eBioscience, #MA5407) at RT for 1 h (all sourced from eBioscience). Slides were washed twice with PBS then incubated with 3% H_2_O_2_ for 30 min. Subsequently, slides were incubated with either a biotinylated goat anti-hamster (1:500, eBioscience, #PA132045) or a donkey anti-rat secondary antibody (1:500, eBioscience, #A18739) for 1 h at RT. Slides were washed twice with PBS. Immunoreactivity was visualized by the avidin-biotin complex method (Vectastain Avidin-Biotin Complex Kit, Vector Laboratories, #PK-4000) and developed for 10 min with diaminobenzidine (DAB). Slides were counterstained with eosin, dehydrated sequentially in graded ethanol (70%, 95% and 100%), immersed in HistoChoice and coverslippped with Permount. A single random representative image of the ischaemic core, undamaged ipsilateral tissue, and contralateral tissue was taken using the 20X objective lens (Olympus BX51 microscope). Three random images were taken in the peri-infarct zone (Supplemental Fig. 3). All images were quantified using ImageJ in a blinded manner (Schneider et al., 2012). CD3^+^ and FoxP3+ cells were counted manually. Total ICAM-1 vessel length (μm) was quantified and then expressed per mm^2^.

### 2.10 Immunofluorescence (Iba-1)

Immunofluorescent analysis of microglia was performed on 10 mice per group. Sections were fixed with 2% paraformaldehyde (Sigma-Aldrich, #HT501128) for 1 h, dried, and washed with 0.5% Triton (Sigma-Aldrich, #X100)/PBS. Slides were incubated in blocking solution (5% BSA/20% Goat Serum (Vector Laboratories, #S-1000) in 0.5% Triton/PBS)) for 30 min. Sections were stained with primary antibodies against Iba-1 (1:500) (Wako, #019–19741) for 2 h. Slides were washed and incubated with goat anti-rabbit Alexa Fluor 488 (1:500)(eBioscience, #A11008) secondary antibody for 1 h, washed and cover-slipped with Vectashield (Vector Laboratories, #H-1200). A single representative image of the ischaemic core, undamaged ipsilateral tissue, and contralateral tissue was taken using the 20X objective lens (Olympus BX51 microscope). Three representative images of the peri-infarct zone were also taken (Supplemental Fig. 4). Positively stained cells were counted using ImageJ (Schneider et al., 2012). Microglia were differentiated into “activated” and “quiescent” states based on morphology (amoeboid or ramified).

### 2.11 Determination of TGF-β1 in spleen by ELISA

Samples of spleen tissue (approximately 20 mg) were frozen in isopentane (−42°C) and stored at −80°C. Spleen (10–40 mg) was processed by homogenisation in cold RIPA buffer containing HALT protease inhibitor cocktail (Thermofisher Scientific, #78429) at 30 μl per 1 mg tissue using a Potter-Elvehjem tissue grinder (PTFE pestle). Lysates were centrifuged at 12,000 rpm for 20 min at 4°C, supernatants were collected and stored at −80°C. The quantification of TGF-β was conducted according to the instructions provided with the TGFβ-1 Mouse ELISA Kit (Invitrogen, #BMS608-4) with samples and standards run in duplicate. Prior to analysis, samples were diluted in assay buffer, activated with 1 M HCl for 1 h and further diluted in assay buffer (final dilution 1:30). Samples were incubated with Biotin Conjugation Solution (100 μl) for 1 h. For visualisation, samples were incubated with TMB substrate (100 μl) for 30 min in the dark. The reaction was halted with Stop Solution and the plate was read on a Wallac Victor2 Multilabel plate reader (PerkinElmer) at 450 nm followed by correction at 620 nm. Total protein concentration (mg/ml) was quantified using a Pierce BCA protein assay kit (Thermofisher Scientific, #23227) according to manufacturer's instructions. Samples were diluted 1:5 in distilled water, and BSA standards were prepared in 20% RIPA buffer. Samples and standards were run in technical duplicate. TGF-β1 concentration (ng/ml) was calculated from the standard curve and expressed as ng/mg total protein.

### 2.12 Statistical analysis

The Kolmogorov–Smirnov test was used to ascertain that the distribution of all data was normal. Flow cytometric and immunohistochemical data are displayed as 10–90 percentile box-and-whisker plots. Two-sided, independent-samples T tests were employed to investigate differences between two groups. Two-way repeated measures ANOVA followed by post-hoc Tukey's test for multiple comparisons was used to investigate differences between groups when the effect of two independent variables (e.g., time, treatment) were studied. A *p*-value < 0.05 was considered significant. Statistical analysis was performed using GraphPad Prism 8.0. All investigators were blinded to treatment groups throughout data acquisition and analysis.

## 3 Results

### 3.1 rPSG1-Fc significantly improves neurobehavioural recovery post-pMCAO without impacting infarct size

The effect of rPSG1-Fc on functional recovery was assessed by determining whether the treatment improved grid-walking test performance post-ischaemia as compared to saline control. All mice underwent testing pre-surgery to establish baseline scores. The grid-walking test was then repeated at days 3 and 5 post-pMCAO.

**Fig. 1:**
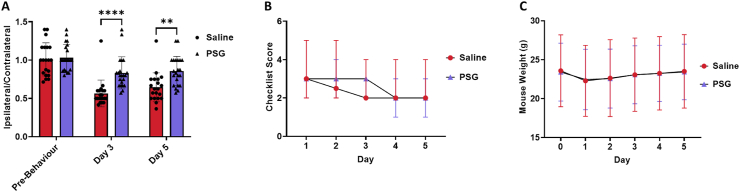
Comparison of A) ipsilateral/contralateral foot fault ratio as assessed by grid-walking test (t = 3, 5 days), B) total checklist score, and C) mouse weight between saline (n = 20) and rPSG1-Fc-treated (n = 22) mice. Two-way repeated measures ANOVA followed by post-hoc Tukey's multiple comparisons was used to investigate differences between groups (* = *p*<0.05, ** = *p*<0.01, *** = *p*<0.001 as compared to saline). Scatter plots depict mean ± standard deviation.

**Fig. 2:**
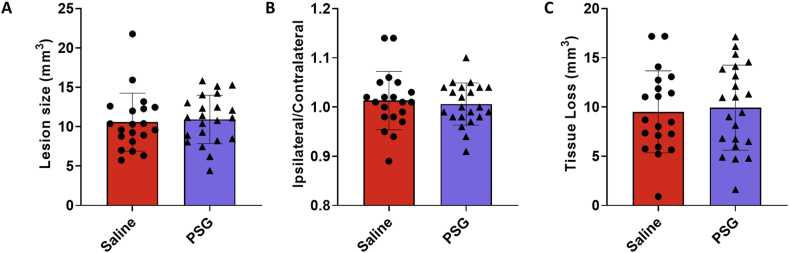
Comparison of A) lesion size (mm^3^), B) ipsilateral/contralateral hemispheric volume ratio, and C) tissue loss (mm^3^) between saline (n = 20) and rPSG1-Fc-treated (n = 22) mice as quantified by NeuN staining (t = 5 days). Two-sided, independent-samples T-tests was used to investigate differences between groups. Scatter plots depict mean ± standard deviation.

In both saline and rPSG1-Fc-treated mice, a reduction in the ipsilateral/contralateral foot fault ratio was observed at both days 3 (saline: *p* <0.0001, rPSG1-Fc: *p* <0.0001) and 5 (saline: *p* = 0.0007, rPSG1-Fc: *p* = 0.0031) post-pMCAO (Fig. 1). When comparing treatment groups, no significant difference was noted between saline and rPSG1-Fc-treated mice at baseline (*p* >0.9999). However, rPSG1-Fc treatment resulted in highly significant improvements in the ipsilateral/contralateral foot fault ratio at both days 3 (*p* <0.0001) and 5 *(p* = 0.0015) post-ischaemia. No significant change was observed between saline and rPSG1-Fc-treated mice at any timepoint in checklist score *(p* = 0.9845) or mouse weight *(p* = 0.7916) (Fig. 2). A beneficial effect of time since ischaemia onset on recovery was confirmed for both measurements (checklist score: *p* <0.0001, mouse weight *p* <0.0001).

The effect of rPSG1-Fc on histological stroke outcome was measured at day 5 post-ischaemia (Fig. 2). In contrast to the impact of treatment on functional recovery, there was no significant difference in lesion size compared to saline-treated mice (*p* = 0.7344). Likewise, no effect on either tissue loss (*p* = 0.7459) or the ratio of ipsilateral/contralateral hemisphere volume (*p* = 0.6571) was observed.

### 3.2 rPSG1-Fc increases circulating CD4^+^ CD25^+^ and CD4^+^ FoxP3+ cell frequencies but does not increase Treg frequency in the periphery post-pMCAO

Based on previous research suggesting that rPSG1-Fc treatment may increase Treg frequency, coupled with the known neuroprotective effects of this lymphocyte subpopulation post-pMCAO, the effect of rPSG1-Fc on peripheral Tregs (defined as CD4^+^ CD25^+^ FoxP3+ cells) was investigated in blood, spleen, draining (cervical) and non-draining lymph nodes (inguinal), and Peyer's patches harvested from mice at 5 days post-ischaemia (Zamorina et al., 2016; Liesz and Kleinschnitz, 2016).

**Fig. 3:**
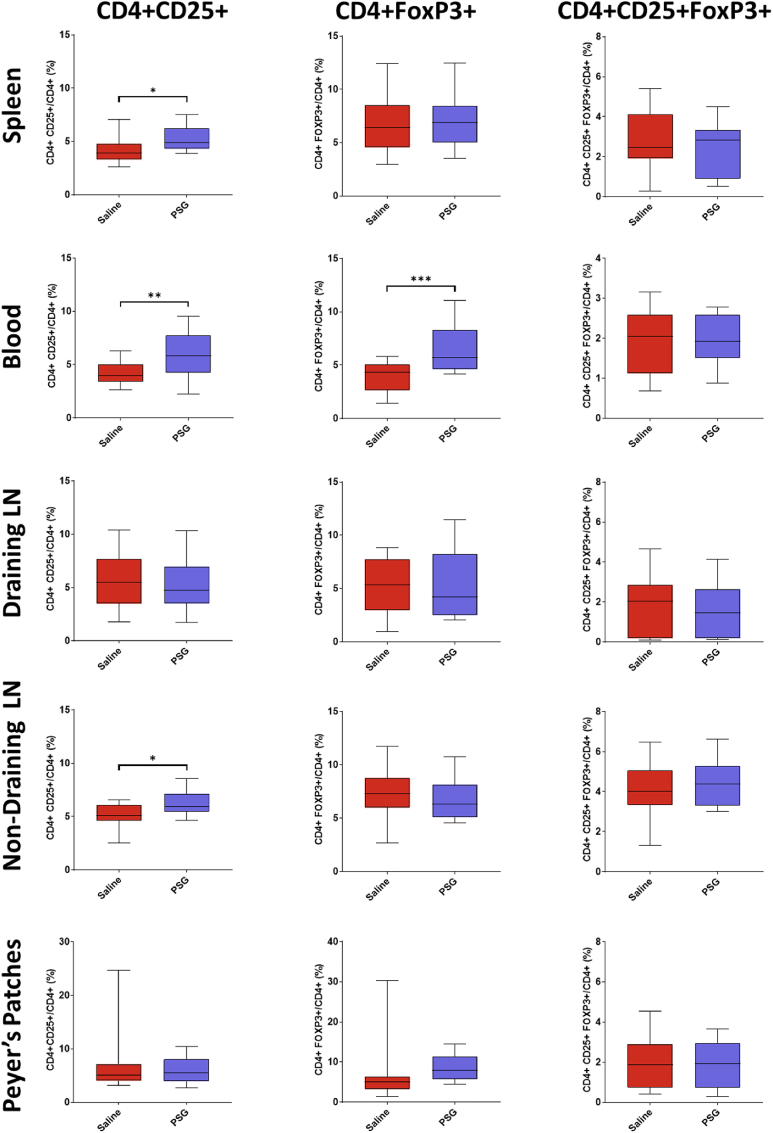
Frequency of CD4^+^ CD25^+^, CD4^+^ FoxP3+, and CD4^+^ CD25^+^ FoxP3+ cells in blood and secondary lymphoid tissue in response to either saline (n = 20) or rPSG1-Fc (n = 22) treatment post-pMCAO (t =5 days). Two-sided, independent-samples T-tests used to investigate differences between groups (* = *p*<0.05, ** = *p*<0.01, *** = *p*<0.001 as compared to saline). Box-and-whisker plots exhibit 10–90 percentiles.

rPSG1-Fc did not increase the frequency of Tregs in any tissues (Fig. 3) (Supplemental Fig. 5). rPSG1-Fc increased the frequency of CD4^+^ CD25^+^ cells in spleen (*p* = 0.0140), blood (*p* = 0.0060), and non-draining lymph nodes (*p* = 0.0122). PSG1-Fc likewise increased CD4^+^ FoxP3+ frequency in blood (*p* = 0.0002).

### 3.3 rPSG1-Fc increases FoxP3+ cell infiltration into the brain post-pMCAO

**Fig. 4:**
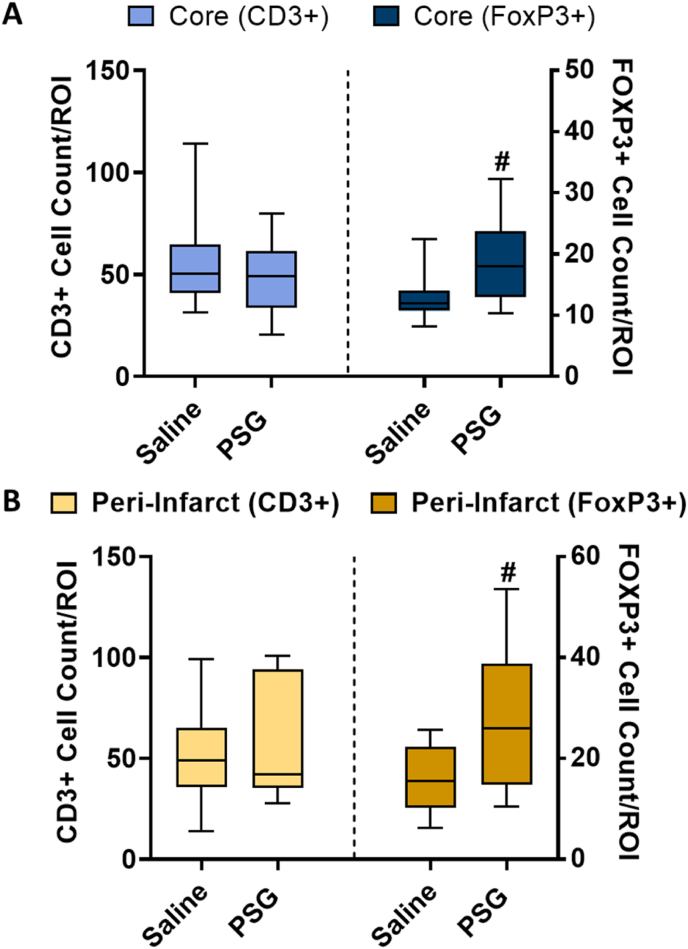
Total CD3^+^ and FoxP3+ cell counts in the infarct core (A) and peri-infarct zone (B) of saline-treated and rPSG1-Fc -treated mice post-pMCAO (t = 5 days) (n = 10 per group). DAB-based immunohistochemistry was used to determine cell counts. Two-sided, independent-samples T-tests or Mann-Whitney tests were used to investigate differences between groups. For comparisons between CD3^+^ groups (* = *p*<0.05, ** = *p*<0.01, *** = *p*<0.001 as compared to saline). For comparison between FoxP3+ groups (# = *p*<0.05, ## = *p*<0.01, ### = *p*<0.001 as compared to saline). Box and whisker plots display the 90/10 percentile at the whiskers, the 75/25 percentiles at the boxes, and the median in the centre line.

The results above suggest that although a 100 μg dose of rPSG1-Fc is not sufficient to increase peripheral CD4^+^ CD25^+^ FoxP3+ Treg frequency post-ischaemia, rPSG1-Fc may promote Treg-like phenotypes in specific tissues and/or enhance anti-inflammatory cytokine secretion. In order to test both hypotheses, we next assessed the effects of PSG1-Fc on brain-infiltrating Tregs, and on T cell cytokine production (next section). CD3^+^ and FoxP3+ cells were counted in the ischaemic core (Fig. 4A) and peri-infarct (Fig. 4B) regions. While no change in CD3^+^ cell counts were observed in either region (*p* = 0.4278, *p* = 0.5347 respectively), rPSG1-Fc treatment produced a significant increase in FoxP3+ cells in both regions (core: *p* = 0.0263, peri-infarct: *p* = 0.0324). This mirrored an increased number of FoxP3+ cells in blood (*p* = 0.0015) (Supplemental Fig. 6).

### 3.4 rPSG1-Fc increases secretion of IL-10 and downregulates production of IFN-γ and IL-17

As rPSG1-Fc increased FoxP3 expression in blood and brain, and as FoxP3 acts as the master switch of regulatory activity, we postulated that rPSG1-Fc may improve recovery post-pMCAO by skewing the T cell phenotype from pro-inflammatory subsets such as Th1 and Th17 towards an anti-inflammatory subset such as Treg. In order to investigate this hypothesis, the expression of interferon gamma (Th1), IL-17 (Th17), and IL-10 (Treg) was quantified in blood and secondary lymphoid tissue via flow cytometric analysis.

**Fig. 5:**
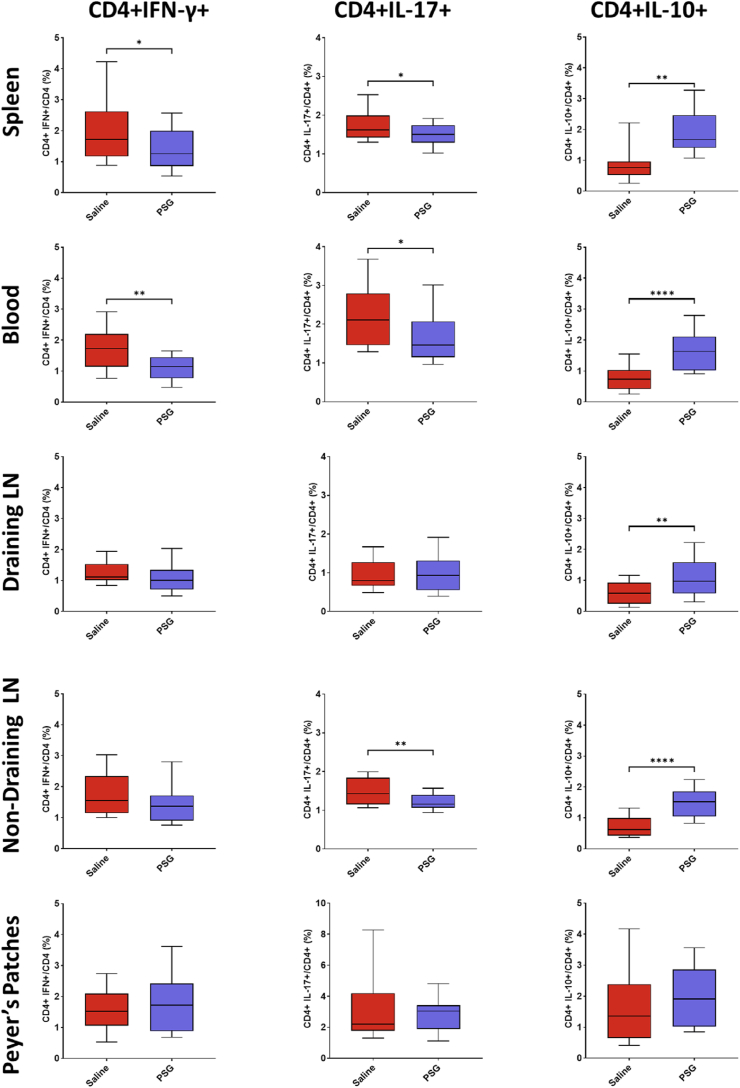
Frequency of CD4^+^ IFNγ+, CD4^+^ IL-17+, and CD4^+^ IL-10+ cells in blood and secondary lymphoid tissue in response to either saline (n = 20) or rPSG1-Fc (n = 22) treatment post-pMCAO (t =5 days). Two-sided, independent-samples T-tests were used to investigate differences between groups (* = *p*<0.05, ** = *p*<0.01, *** = *p*<0.001 as compared to saline). Box and whisker plots display the 90/10 percentile at the whiskers, the 75/25 percentiles at the boxes, and the median in the centre line.

PSG1-Fc decreased the production of interferon gamma (IFN-γ) among CD4^+^ cells in spleen (*p* = 0.0356) and blood (*p* = 0.0021) (Fig. 5). Similarly, a reduction in CD4^+^ IL17+ cells was observed in rPSG1-Fc-treated mice in spleen (*p* = 0.0444), blood (*p* = 0.0317), and non-draining lymph nodes (*p* = 0.0090). PSG1-Fc caused an increase in IL-10 production in CD4^+^ cells in spleen (*p* = 0.0011), blood (*p* <0.0001), draining lymph nodes (*p* = 0.0078), and non-draining lymph nodes (*p* <0.0001).

**Fig. 6:**
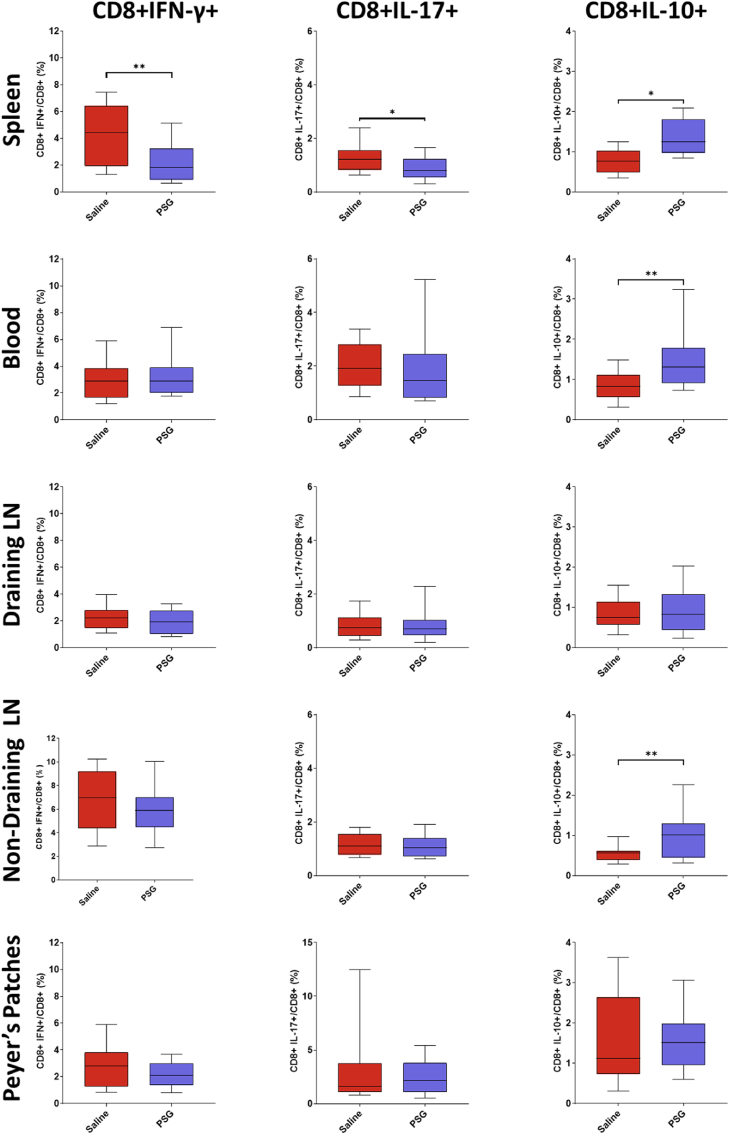
Frequency of CD8^+^ IFNγ+, CD8^+^ IL-17+, and CD8^+^ IL-10+ cells in blood and secondary lymphoid tissue in response to either saline (n = 20) or rPSG1-Fc (n = 22) treatment post-pMCAO (t =5 days). Two-sided, independent-samples T-tests were used to investigate differences between groups (* = *p*<0.05, ** = *p*<0.01, *** = *p*<0.001 as compared to saline). Box and whisker plots display the 90/10 percentile at the whiskers, the 75/25 percentiles at the boxes, and the median in the centre line.

Among CD8^+^ cells (Fig. 6), rPSG1-Fc reduced IFN-γ and IL-17 production in spleen (*p = *0.0068, *p* = 0.0416 respectively) and increased IL-10 in spleen (*p* = 0.0274), blood (*p* = 0.0094), and non-draining lymph nodes (*p* = 0.0067).

Overall, the administration of rPSG1-Fc modulated T cell cytokine production in blood and spleen away from IFN-γ/IL-17 and towards IL-10. However, this result was not observed in draining lymph nodes.

### 3.5 rPSG1-Fc reduces activated microglia in the infarct core of post-ischaemic mice

**Fig. 7:**
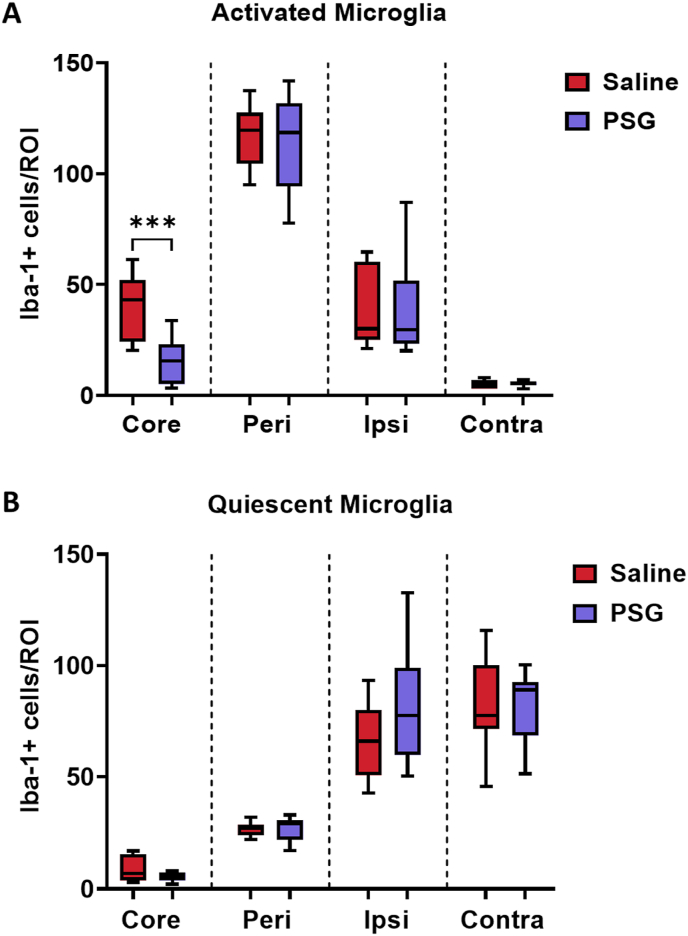
Total numbers of A) activated microglia and B) quiescent microglia in the core, peri-infarct zone, healthy ipsilateral tissue, and contralateral tissue of saline-treated and rPSG1-Fc -treated mice post-pMCAO (t = 5 days) (n = 10 per group). Microglia morphology was determined as “activated” (amoeboid) or “quiescent” (ramified) via Iba-1 immunofluorescent staining. Two-sided, independent-samples T-tests were used to investigate differences between groups (* = p<0.05, ** = p<0.01, *** = p<0.001 compared to saline). Box and whisker plots display the 90/10 percentile at the whiskers, the 75/25 percentiles at the boxes, and the median in the centre line.

Infiltrating Tregs may restrain post-stroke neuroinflammation via a microglia-dependent mechanism (Xie et al., 2014; Cai et al., 2020; Zera and Buckwalter, 2021; Shi et al., 2021). Thus, the effect of rPSG1-Fc on microglial activation was investigated using Iba-1 staining to analyse microglia morphology (Fig. 7).

rPSG1-Fc significantly reduced activated microglia (Iba-1 positive cells with an “amoeboid” morphology) in the infarct core (*p* = 0.0007), with a trend towards reduced numbers of quiescent microglia in the core region (*p* = 0.0583). rPSG1-Fc had no effect on activated microglial cell numbers in the peri-infarct zone (*p* = 0.6767), healthy ipsilateral tissue (*p* = 0.8413), or contralateral tissue (*p* = 0.8875) compared to saline-treated mice. No impact on quiescent microglia (displaying a “ramified” morphology) was noted in these regions (*p* >0.9999, *p* = 0.1504, *p* = 0.9371 respectively).

### 3.6 rPSG1-Fc does not influence ICAM-1 expression in post-pMCAO brain tissue

**Fig. 8:**
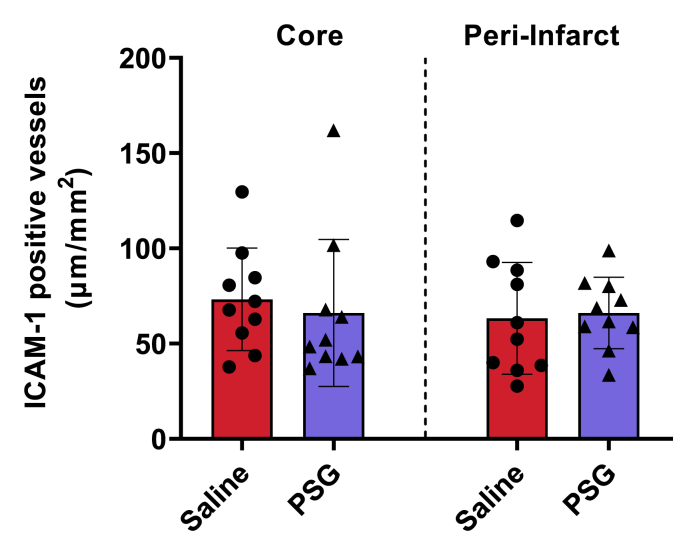
Total ICAM-1 positive vessel length (μm/mm^2^) in the infarct core and peri-infarct zone of saline-treated and rPSG1-Fc -treated mice post-pMCAO (t = 5 days) (n = 10 per group). DAB-based immunohistochemistry was used to determine cell counts. Two-sided, independent-samples T-tests were used to investigate differences between groups (* = *p*<0.05, ** = *p*<0.01, *** = *p*<0.001 compared to saline). Bar plots display the mean±SD. Dots represent individual mouse values.

Based on previous work which identified CD9 (a key regulator of cell adhesion) as a receptor for some mouse PSGs, we postulated that rPSG1-Fc may decrease leukocyte infiltration into the ischaemic brain and thus promote stroke recovery via reduced adhesion molecule expression (Bebo and Dveksler, 2005). As per Fig. 8, however, no significant change in ICAM-1 expression was observed in either core (*p* = 0.6376) or peri-infarct (*p* = 0.8003) tissue.

### 3.7 rPSG1-Fc treatment does not increase TGF-β expression in spleen post-ischaemia

**Fig. 9:**
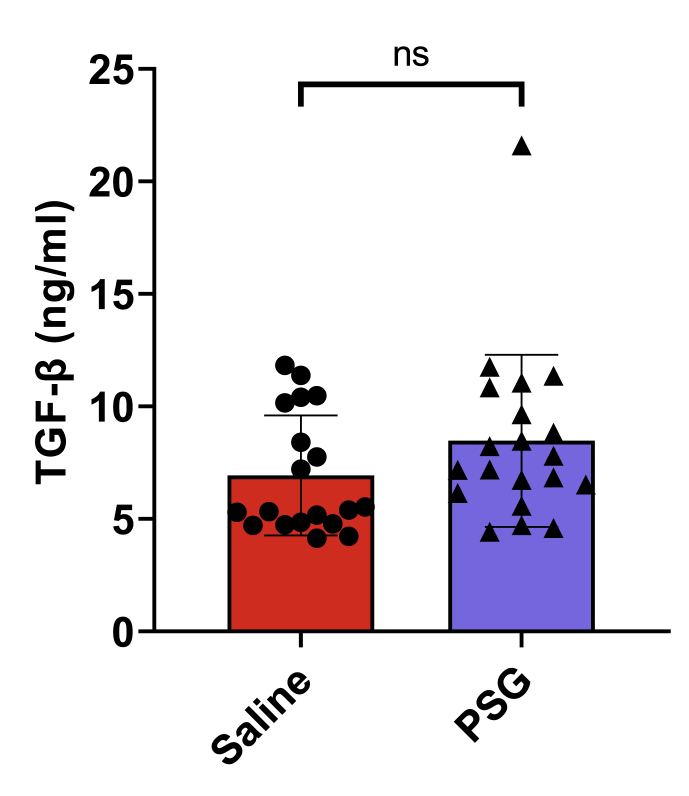
Total TGF-β in the spleens of saline and rPSG1-Fc-treated mice expressed as nanoograms/milligram tissue (normalised for protein content) (t = 5 days) (n = 19–20 per group). Two-sided, independent-samples T-tests were used to investigate differences between groups (* = *p*<0.05, ** = *p*<0.01, *** = *p*<0.001 compared to saline).

Previous research suggests PSG1 activates latent TGF-β (Warren et al., 2018; Ballesteros et al., 2015). Indeed, the effect of PSGs on this cytokine is believed to be responsible for their ability to upregulate Treg cells and drive improvement in inflammatory disease states (Blois et al., 2014; Jones et al., 2016). Therefore, although no increase in peripheral Tregs was observed in this study, the levels of splenic TGF-β1 were determined by ELISA at 5 days post-ischaemia to determine whether an increase in expression of this cytokine may underlie any of the immunomodulatory responses observed in this study. As per Fig. 9 however, no changes in the amount of TGF-β (*p* = 0.4054) in post-ischaemic mouse spleens were observed (*p* = 0.1566).

## 4 Discussion

Pregnancy-specific glycoproteins exhibit a variety of immunomodulatory properties and have been shown to improve recovery in disease models including acute graft-versus-host disease, colitis and arthritis (Moore and Dveksler, 2014; Falcón et al., 2014; Zamorina et al., 2016; Blois et al., 2014; Jones et al., 2016, 2019; Martinez et al., 2013). Specifically, PSG1 increases Tregs, a lymphocyte subpopulation associated with improved ischaemic stroke recovery (Falcón et al., 2014; Blois et al., 2014; Jones et al., 2019; Wu et al., 2021). Here, we determined the effect of an IgG1-Fc tagged recombinant pregnancy-specific glycoprotein-1 (rPSG1-Fc) on ischaemic stroke recovery in mice, using a permanent model of brain ischaemia. Our results demonstrate that rPSG1-Fc treatment significantly improved neurobehavioural recovery at days 3 and 5 post-ischaemia compared to saline-treated controls. However, no impact on histological outcome was observed. In CD4^+^ cells, rPSG1-Fc enhanced secretion of IL-10 in spleen, blood, and draining and non-draining lymph nodes, while downregulating the production of IFN-γ and IL-17 in spleen and blood but not in draining lymph nodes. rPSG1-Fc also reduced CD8^+^ IFN-γ and CD8^+^ IL-17+ cells in spleen, while upregulating CD8^+^ IL-10+ cells in spleen, blood, and non-draining lymph nodes. rPSG1-Fc increased both circulating CD4^+^ FoxP3+ cells and brain-infiltrating FoxP3+ cells, but failed to increase Treg frequencies in any tissue. rPSG1-Fc also reduced activated microglia in the infarct core. Overall, rPSG1-Fc appears a promising candidate stroke immunotherapy, possibly due to a strong enhancing effect on IL-10 secretion.

The lack of effect of rPSG1-Fc on checklist scores or mouse weights may be due to the lack of sensitivity of these measures, as they are only minimally impacted by small cortical lesions obtained in our model. The lack of correlation between the effects of rPSG1-Fc on functional and histological outcomes may seem unexpected. In stroke patients, however, a treatment-induced improvement in behaviour does not necessarily reflect a concomitant reduction in lesion size (Alexander et al., 2010). The early reliance on infarct size as the main experimental readout has been suggested to account, at least in part, for the lack of translation to clinical efficacy (Minnerup et al., 2010; (STAIR) STAIR, 1999), leading to the suggestion that neurobehavioural tests used to detect experimental stroke-induced changes in cognition, mood, and sensorimotor function would be better predictors for clinical efficacy (Balkaya et al., 2018). Future studies of rPSG1-Fc as a potential stroke therapy should therefore prioritise behavioural testing, as recommended by STAIR guidelines, with a focus on long-term prognosis (Fisher et al., 2009).

The finding that rPSG1-Fc improved post-stroke recovery matches results seen with PSG1 in models of colitis, arthritis, and acute graft-versus-host disease (Falcón et al., 2014; Blois et al., 2014; Jones et al., 2019). While these disorders all involve immune and/or inflammatory factors, the exact mechanisms of action of PSG1 in each model may differ. We initially postulated rPSG1-Fc would provide neuroprotection via a Treg-dependent mechanism, based on studies showing that *in vivo* expression of PSG1 enriched CD4^+^ CD25^+^ FoxP3+ cells in spleen (Falcón et al., 2014; Martínez et al., 2012). Similar effects were reported after intraperitoneal administration of 100 μg PSG1 every 2 days (Jones et al., 2019). In our study, rPSG1-Fc did not modulate CD4^+^CD25+Foxp3+ Tregs in any analysed tissue. Increased CD4^+^ FoxP3+ cells however are in line with the increased CD4^+^ FoxP3+ LAP + cells observed in PSG1-treated mice, the heightened levels of FoxP3 mRNA noted among CD4^+^ cells, and the results obtained with isolated mouse and human CD4^+^ cells (Zamorina et al., 2016; Blois et al., 2014; Jones et al., 2019)*.* Our observations are in agreement with effects of other PSG family members such as PSG9 (Jones et al., 2016). Given that CD4^+^ CD25^+^ FoxP3+ cells tripled after ten doses of PSG1 in that study, it is possible that an extended treatment regimen is required to fully expand the Treg compartment (Jones et al., 2019). Conversely, the single dose of rPSG1-Fc employed in this study may have increased Tregs only transiently post-ischaemia. Since elevated Tregs were observed two weeks after PSG1 withdrawal in the ten-dose study, however, it appears more likely a multiple dose regimen is required to produce the known effects of PSG1 on these cells (Jones et al., 2019).

Our observation that rPSG1-Fc treatment reduced levels of both secreted IFN-γ and IL-17, as measured by intracellular cytokine staining, is consistent with several previous studies (Falcón et al., 2014; Motrán et al., 2003; Rayev et al., 2017; Blois et al., 2014). Similarly, increased IL-10 production in several tissues observed here (principally among CD4^+^ cells) matches a wealth of evidence wherein PSG1 is expressed *in vivo* (Falcón et al., 2014; Motrán et al., 2003; Jones et al., 2019), administered as a recombinant protein (Blois et al., 2014; Martínez et al., 2012), and when examined *in vitro* (Ha et al., 2005). In acute ischaemic stroke, low levels of IL-10 predict worse outcomes, while exogenous administration of the cytokine improves recovery in experimental stroke (Garcia et al., 2017). Previous work with other Treg-targeted immunotherapies shows IL-10 plays a central role in Treg-mediated neuroprotection (Liesz et al., 2013; Na et al., 2015). Taken together with the observed effects on FoxP3, the boosted secretion of IL-10 noted in rPSG1-Fc-treated mice may suggest a mechanism wherein PSG enhances regulatory cells which, following infiltration into the infarcted brain, provide neuroprotection via IL-10. Elements of the immune system beyond Tregs are likely involved. rPSG1-Fc reduced microglial activation in this study. Based on the fact that PSGs enhanced IL-10 production among myeloid cells, coupled with the known links between Tregs and microglia in post-stroke repair, a more complex mechanism involving modulation of microglia phenotype by rPSG1-Fc is also possible (Motrán et al., 2003; Ha et al., 2005; Xie et al., 2014; Cai et al., 2020). However, further data on the impact of PSG on M1-like and M2-like phenotypes is required.

The differential effect of rPSG1-Fc on the secretion of IL-10 among CD8^+^ cells in spleen, blood, and non-draining lymph nodes (where increases were noted) versus the draining cervical lymph nodes (where no change was observed) requires further study. Similarly, rPSG1-Fc reduced CD4^+^ IL-17+ and CD4^+^ IFN-γ T cells in spleen and blood but not draining lymph nodes. One possible explanation may involve the intimate connection between these draining lymph nodes and the brain. Even under non-stroke conditions, the meningeal lymphatic system facilitates the drainage of cerebrospinal fluid macromolecules to the cervical lymph nodes (Da Mesquita et al., 2018). Post-stroke, such “brain-cervical lymph nodes crosstalk” systems are crucially involved in the clearance of neurotoxic material from the infarcted brain (Bolte and Lukens, 2021). Recently, Esposito et al., identified that this same lymphatic fluid can activate macrophages (and likely other immune cells) in the draining lymph nodes as early as 24 h post-ischaemia (Esposito et al., 2019). Therefore, it is possible that in the spleen and blood, rPSG1-Fc has the capacity to shift the immune response away from IFN-γ/IL-17 and towards IL-10 as observed in other studies. But in the draining lymph node, the tolerizing signal of rPSG1-Fc is cancelled out by the acute pro-inflammatory signalling described above.

Analysis of spleen TGF-β1 levels revealed no significant differences between rPSG1-Fc and saline-treated mice. Previous evidence shows PSGs increase free levels of this cytokine in autoimmune disease models (Falcón et al., 2014) and in the steady state (Ha et al., 2005; Warren et al., 2018; Blois et al., 2014; Ballesteros et al., 2015; Jones et al., 2016). However, most studies associating PSGs with increased TGF-β1 were conducted with cell lines, protein systems, or *in vivo* expression models, as opposed to subcutaneous injection employed here (Falcón et al., 2014; Ha et al., 2005; Warren et al., 2018; Ballesteros et al., 2015; Jones et al., 2016; Martínez et al., 2012). In the two studies where PSG1 was administered directly via intraperitoneal injection, TGF-β1 levels were not directly determined (Blois et al., 2014; Jones et al., 2019). Indeed, only the downstream hallmarks of increased TGF-β1-signalling (elevated FoxP3, increased Tregs, upregulated IL-10 signalling, and higher SMAD2/3 phosphorylation) were demonstrated. In the current study, TGF-β1 levels could only be quantified in the spleen at 5 days post-ischaemia. It is possible that TGF-β1 may have been sequestrated in insoluble extracellular matrix, and that the use of serum, lymph nodes or brain may have yielded different results (Khan et al., 2012). Given the ELISA assay employed in this study measured total TGF-β levels, and not the active or latent form independently, it is also possible the activating effect of rPSG1-Fc on latent TGF-β previously described was not accurately captured (Warren et al., 2018).

The relative strengths of this study include appropriate sample sizes determined by *a priori* power calculation, use of both male and female mice, and behavioural outcome in a permanent model of brain ischaemia which more accurately mirrors the clinically observed low reperfusion rates (Gauberti et al., 2021; Bowler et al., 1998). We demonstrate the *in* vivo effect of rPSG1-Fc in ischaemic stroke with a clear impact on the cytokine-secreting phenotypes of CD4^+^ and CD8^+^ T cells. While this study provides preliminary evidence for several potential mechanisms of action for rPSG1-Fc in stroke, it should be emphasized that this is only evidence of association and not causation. Another limitation of this study is that the impact of PSG1-Fc in post-stroke mice was only examined at one timepoint. However, the selected treatment regimen was based on experience with rPSG1-Fc in other disease models and, more generally, with other putative Treg-targeted stroke immunotherapies (Blois et al., 2014; Na et al., 2015; Zhang et al., 2018). Further prospective studies are required to fully determine which aspects of the immune system play a definitive causative role.

## 5 Conclusion

Pregnancy-specific glycoproteins (PSGs) have garnered recent interest for the treatment of a number of inflammatory conditions, principally due to their observed effects on regulatory immune cells such as Tregs and anti-inflammatory cytokines (e.g. TGF-β, IL-10) (Martinez et al., 2013). This well-powered study provides strong evidence that a recombinant form of PSG1 improves functional recovery in a permanent model of brain ischaemia, without impacting infarct size. We identify a possible mechanism through enhanced secretion of IL-10 among circulating CD4^+^ cells, and decreased expression of IFN-γ and IL-17, suggesting an overall modulation towards a suppressive phenotype. Reduced microglial activation supports this hypothesis. The apparent lack of an effect on TGF-β levels in this study requires further investigation (Zera and Buckwalter, 2021). In order to confirm whether IL-10 and/or Tregs play a role in the neuroprotective effect of rPSG1-Fc, it should now be tested in mice in which IL-10 or Tregs are blocked or depleted (Pérez-de Puig et al., 2013; Walsh et al., 2014). Confirmation of dose and treatment duration needed to confer maximal benefit is also required. The potential adverse effects of rPSG1-Fc treatment in stroke also must be elucidated. rPSG1-Fc may lack the toxicity and immunogenicity observed with other stroke immunotherapies (as suggested by high levels of the protein found during human pregnancy and its expression in gastrointestinal tract epithelium) (Houston et al., 2016). But while the absence of drug-induced lymphopenia would reduce the risk of treatment-associated infection, it should be recognised that pregnancy itself is associated with increased risks for certain types of infections, and that the immunological changes behind this trend (e.g., increased Treg-derived IL-10) could be conferred by rPSG1-Fc treatment (Houston et al., 2016; Zhang et al., 2021; Abu-Raya et al., 2020; Rowe et al., 2011). Finally, the possibility to combine rPSG1-Fc with currently available methods of recanalization (i.e., tPA, thrombectomy) should be investigated, especially given the expanding indications for such interventions (Sporns et al., 2021). Overall, this study provides the first insight into the effect of rPSG1-Fc in acute ischaemic stroke, alongside potential mechanisms of action.

## Consent for publication

Not applicable.

## Availability of data and materials

The data that supports the findings of this study are available from the corresponding author upon reasonable request.

## Funding

This work was supported by a Government of Ireland Postgraduate Scholarship from the 10.13039/501100002081Irish Research Council (grant number GOIPG/2017/431), the Interreg Atlantic Area Programme, EAPA_791/2018, 2019–21), and a 10.13039/501100001602Science Foundation Ireland Grant (BIAP2015).

## Authors' contributions

KM, AM, TM and CW conceived and directed the project. JS conducted animal surgery. KM carried out behavioural studies. JS prepared histological sections. KM carried out flow cytometry and *in vitro* experiments and interpreted results. JMW prepared PSG1-Fc protein. KM, AM, TM and CW wrote the manuscript. All authors gave final approval for the submitted version.

## Declaration of competing interest

Tom Moore and Christian Waeber are named on a patent application on use of PSG proteins in treatment of neurological disorders including stroke. We do not declare any other competing interests related to this work. We can confirm that all authors have approved the manuscript for submission and the contents of the manuscript have not been published or submitted elsewhere for publication.

## References

[bib1] Abu-Raya B., Michalski C., Sadarangani M., Lavoie P.M. (2020). Maternal immunological adaptation during normal pregnancy. Front. Immunol..

[bib2] Alexander L.D., Black S.E., Gao F., Szilagyi G., Danells C.J., McIlroy W.E. (2010). Correlating lesion size and location to deficits after ischemic stroke: the influence of accounting for altered peri-necrotic tissue and incidental silent infarcts. Behav. Brain Funct..

[bib3] Balkaya M.G., Trueman R.C., Boltze J., Corbett D., Jolkkonen J. (2018). Behavioral outcome measures to improve experimental stroke research. Behav. Brain Res..

[bib4] Ballesteros A., Mentink-Kane M.M., Warren J., Kaplan G.G., Dveksler G.S. (2015). Induction and activation of latent transforming growth factor-β1 are carried out by two distinct domains of pregnancy-specific glycoprotein 1 (PSG1). J. Biol. Chem..

[bib5] Bebo B.F., Dveksler G.S. (2005). Evidence that pregnancy specific glycoproteins regulate T-Cell function and inflammatory autoimmune disease during pregnancy. Curr. Drug Targets - Inflamm. Allergy.

[bib6] Blois S.M., Sulkowski G., Tirado-González I., Warren J., Freitag N., Klapp B.F. (2014). Pregnancy-specific glycoprotein 1 (PSG1) activates TGF-β and prevents dextran sodium sulfate (DSS)-induced colitis in mice. Mucosal Immunol..

[bib7] Bolte A.C., Lukens J.R. (2021). Neuroimmune cleanup crews in brain injury. Trends Immunol..

[bib8] Bowler J.V., Wade J.P., Jones B.E., Nijran K.S., Steiner T.J. (1998). Natural history of the spontaneous reperfusion of human cerebral infarcts as assessed by 99mTc HMPAO SPECT. J. Neurol. Neurosurg. Psychiatry.

[bib9] Cai Y., Xu T.T., Lu C.Q., Ma Y.Y., Chang D., Zhang Y. (2020). Endogenous regulatory T cells promote M2 macrophage phenotype in diabetic stroke as visualized by optical imaging. Transl Stroke Res.

[bib10] Da Mesquita S., Fu Z., Kipnis J. (2018). The meningeal lymphatic system: a new player in neurophysiology. Neuron.

[bib11] Diaz Diaz A.C., Malone K., Shearer J.A., Moore A.C., Waeber C. (2021). Histological, behavioural and flow cytometric datasets relating to acute ischaemic stroke in young, aged and ApoE. Data Brief.

[bib12] Esposito E., Ahn B.J., Shi J., Nakamura Y., Park J.H., Mandeville E.T. (2019). Brain-to-cervical lymph node signaling after stroke. Nat. Commun..

[bib13] Falcón C.R., Martínez F.F., Carranza F., Cervi L., Motrán C.C. (2014). In vivo expression of recombinant pregnancy-specific glycoprotein 1a inhibits the symptoms of collagen-induced arthritis. Am. J. Reprod. Immunol..

[bib14] Fisher M., Feuerstein G., Howells D.W., Hurn P.D., Kent T.A., Savitz S.I. (2009). Update of the stroke therapy academic industry roundtable preclinical recommendations. Stroke.

[bib15] Ganesh A., Goyal M. (2018). Thrombectomy for acute ischemic stroke: recent insights and future directions. Curr. Neurol. Neurosci. Rep..

[bib16] Garcia J.M., Stillings S.A., Leclerc J.L., Phillips H., Edwards N.J., Robicsek S.A. (2017). Role of interleukin-10 in acute brain injuries. Front. Neurol..

[bib17] Gauberti M., Martinez de Lizarrondo S., Vivien D. (2021). Thrombolytic strategies for ischemic stroke in the thrombectomy era. J. Thromb. Haemostasis.

[bib18] Ha C.T., Waterhouse R., Wessells J., Wu J.A., Dveksler G.S. (2005). Binding of pregnancy-specific glycoprotein 17 to CD9 on macrophages induces secretion of IL-10, IL-6, PGE2, and TGF-beta1. J. Leukoc. Biol..

[bib19] Houston A., Williams J.M., Rovis T.L., Shanley D.K., O'Riordan R.T., Kiely P.A. (2016). Pregnancy-specific glycoprotein expression in normal gastrointestinal tract and in tumors detected with novel monoclonal antibodies. mAbs.

[bib20] Johnson W., Onuma O., Owolabi M., Sachdev S. (2016). Stroke: a global response is needed. Bull. World Health Organ..

[bib21] Jones K., Ballesteros A., Mentink-Kane M., Warren J., Rattila S., Malech H. (2016). PSG9 stimulates increase in FoxP3+ regulatory T-cells through the TGF-β1 pathway. PLoS One.

[bib22] Jones K., Bryant S., Luo J., Kiesler P., Koontz S., Warren J. (2019). Recombinant pregnancy-specific glycoprotein 1 has a protective role in a murine model of acute graft-versus-host disease. Biol. Blood Marrow Transplant..

[bib23] Khan S.A., Joyce J., Tsuda T. (2012). Quantification of active and total transforming growth factor-β levels in serum and solid organ tissues by bioassay. BMC Res. Notes.

[bib24] Lee J.A., Spidlen J., Boyce K., Cai J., Crosbie N., Dalphin M. (2008). MIFlowCyt: the minimum information about a flow cytometry experiment. Cytometry.

[bib25] Liesz A., Kleinschnitz C. (2016). Regulatory T cells in post-stroke immune homeostasis. Transl Stroke Res..

[bib26] Liesz A., Zhou W., Na S.Y., Hämmerling G.J., Garbi N., Karcher S. (2013). Boosting regulatory T cells limits neuroinflammation in permanent cortical stroke. J. Neurosci..

[bib27] Llovera G., Roth S., Plesnila N., Veltkamp R., Liesz A. (2014). Modeling stroke in mice: permanent coagulation of the distal middle cerebral artery. JoVE.

[bib28] Malone K., Amu S., Moore A.C., Waeber C. (2018). The immune system and stroke: from current targets to future therapy. Immunol. Cell Biol..

[bib29] Malone K., Amu S., Moore A.C., Waeber C. (2019). Immunomodulatory therapeutic strategies in stroke. Front. Pharmacol..

[bib30] Martínez F.F., Knubel C.P., Sánchez M.C., Cervi L., Motrán C.C. (2012). Pregnancy-specific glycoprotein 1a activates dendritic cells to provide signals for Th17-, Th2-, and Treg-cell polarization. Eur. J. Immunol..

[bib31] Martinez F.F., Cervi L., Knubel C.P., Panzetta-Dutari G.M., Motran C.C. (2013). The role of pregnancy-specific glycoprotein 1a (PSG1a) in regulating the innate and adaptive immune response. Am. J. Reprod. Immunol..

[bib32] Masson G.M., Anthony F., Wilson M.S. (1983). Value of Schwangerschaftsprotein 1 (SP1) and pregnancy-associated plasma protein-A (PAPP-A) in the clinical management of threatened abortion. Br. J. Obstet. Gynaecol..

[bib33] Minnerup J., Wersching H., Brokinkel B., Dziewas R., Heuschmann P.U., Nabavi D.G. (2010). The impact of lesion location and lesion size on poststroke infection frequency. J. Neurol. Neurosurg. Psychiatry.

[bib34] Moore T., Dveksler G.S. (2014). Pregnancy-specific glycoproteins: complex gene families regulating maternal-fetal interactions. Int. J. Dev. Biol..

[bib35] Moore T., Williams J.M., Becerra-Rodriguez M.A., Dunne M., Kammerer R., Dveksler G. (2022). Pregnancy-specific glycoproteins: evolution, expression, functions and disease associations. Reproduction.

[bib36] Motrán C.C., Díaz F.L., Gruppi A., Slavin D., Chatton B., Bocco J.L. (2002). Human pregnancy-specific glycoprotein 1a (PSG1a) induces alternative activation in human and mouse monocytes and suppresses the accessory cell-dependent T cell proliferation. J. Leukoc. Biol..

[bib37] Motrán C.C., Diaz F.L., Montes C.L., Bocco J.L., Gruppi A. (2003). In vivo expression of recombinant pregnancy-specific glycoprotein 1a induces alternative activation of monocytes and enhances Th2-type immune response. Eur. J. Immunol..

[bib38] Na S.Y., Mracsko E., Liesz A., Hünig T., Veltkamp R. (2015). Amplification of regulatory T cells using a CD28 superagonist reduces brain damage after ischemic stroke in mice. Stroke.

[bib39] Percie du Sert N.H.V., Ahluwalia A., Alam S., Avey M.T., Baker M. (2020). The ARRIVE guidelines 2.0: updated guidelines for reporting animal research. PLoS Biol..

[bib40] Pérez-de Puig I., Miró F., Salas-Perdomo A., Bonfill-Teixidor E., Ferrer-Ferrer M., Márquez-Kisinousky L. (2013). IL-10 deficiency exacerbates the brain inflammatory response to permanent ischemia without preventing resolution of the lesion. J. Cerebr. Blood Flow Metabol..

[bib41] Rayev M.B., Litvinova L.S., Yurova K.A., Dunets N.A., Khaziakhmatova O.G., Timganova V.P. (2017). Role of the pregnancy-specific glycoprotein in regulation of the cytokine and chemokine profiles of intact mononuclear cells. Dokl. Biol. Sci..

[bib42] Rowe J.H., Ertelt J.M., Aguilera M.N., Farrar M.A., Way S.S. (2011). Foxp3(+) regulatory T cell expansion required for sustaining pregnancy compromises host defense against prenatal bacterial pathogens. Cell Host Microbe.

[bib43] Schaar K.L., Brenneman M.M., Savitz S.I. (2010). Functional assessments in the rodent stroke model. Exp. Transl. Stroke Med..

[bib44] Schneider C.A., Rasband W.S., Eliceiri K.W. (2012). NIH Image to ImageJ: 25 years of image analysis. Nat. Methods.

[bib45] Shi L., Sun Z., Su W., Xu F., Xie D., Zhang Q. (2021). Treg cell-derived osteopontin promotes microglia-mediated white matter repair after ischemic stroke. Immunity.

[bib46] Silver R.M., Heyborne K.D., Leslie K.K. (1993). Pregnancy specific beta 1 glycoprotein (SP-1) in maternal serum and amniotic fluid; pre-eclampsia, small for gestational age fetus and fetal distress. Placenta.

[bib47] Sporns P.B., Fiehler J., Ospel J., Safouris A., Hanning U., Fischer U. (2021). Expanding indications for endovascular thrombectomy-how to leave no patient behind. Ther Adv Neurol Disord.

[bib48] STAIR (1999). Recommendations for standards regarding preclinical neuroprotective and restorative drug development. Stroke.

[bib49] Walsh J.T., Zheng J., Smirnov I., Lorenz U., Tung K., Kipnis J. (2014). Regulatory T cells in central nervous system injury: a double-edged sword. J. Immunol..

[bib50] Wang H., Wang Z., Wu Q., Yuan Y., Cao W., Zhang X. (2021). Regulatory T cells in ischemic stroke. CNS Neurosci. Ther..

[bib51] Warren J., Im M., Ballesteros A., Ha C., Moore T., Lambert F. (2018). Activation of latent transforming growth factor-β1, a conserved function for pregnancy-specific beta 1-glycoproteins. Mol. Hum. Reprod..

[bib52] Wu Y., Li J., Shou J., Zhang W., Chen C. (2021). Diverse functions and mechanisms of regulatory T cell in ischemic stroke. Exp. Neurol..

[bib53] Xie L., Sun F., Wang J., Mao X., Yang S.H., Su D.M. (2014). mTOR signaling inhibition modulates macrophage/microglia-mediated neuroinflammation and secondary injury via regulatory T cells after focal ischemia. J. Immunol..

[bib54] Zamorina S.A., Litvinova L.S., Yurova K.A., Dunets N.A., Khaziakhmatova O.G., Timganova V.P. (2016). The effect of pregnancy-specific β1-glycoprotein 1 on the transcription factor FOXP3 expression by immunocompetent cells. Dokl. Biochem. Biophys..

[bib55] Zera K.A., Buckwalter M.S. (2021). T cells direct microglial repair of white matter after stroke. Trends Neurosci..

[bib56] Zhang H., Xia Y., Ye Q., Yu F., Zhu W., Li P. (2018). Expansion of regulatory T cells with IL-2/IL-2 antibody complex protects against transient ischemic stroke. J. Neurosci..

[bib57] Zhang D., Ren J., Luo Y., He Q., Zhao R., Chang J. (2021). T cell response in ischemic stroke: from mechanisms to translational insights. Front. Immunol..

