# Supplementary material for: Recombinant pregnancy-specific glycoprotein-1-Fc reduces functional deficit in a mouse model of permanent brain ischaemia
Source: Brain Behav Immun Health. 2022 Aug 24;25:100497. doi: 10.1016/j.bbih.2022.100497 (PMC9475273; doi:10.1016/j.bbih.2022.100497)
Supplement: Supplementary Figure 4 [file mmc4.docx]

**Supplementary Figure 4**

**Article Title**

Recombinant pregnancy-specific glycoprotein-1-Fc reduces functional deficit in a mouse model of permanent brain ischaemia

**Journal**

Brain, Behaviour, and Immunity

**Authors**

Kyle Malone1,2, Jennifer A Shearer1,2, John M Williams3, Anne C Moore3, Tom Moore3*, Christian Waeber1,2*

**Affiliations**

^1^Department of Pharmacology and Therapeutics, Western Gateway Building, University College Cork, Cork, Ireland

^2^School of Pharmacy, University College Cork, Cork, Ireland

^3^School of Biochemistry and Cell Biology, University College Cork, Cork, Ireland.

**Corresponding Author Email**

Kyle.malone@ucc.ie


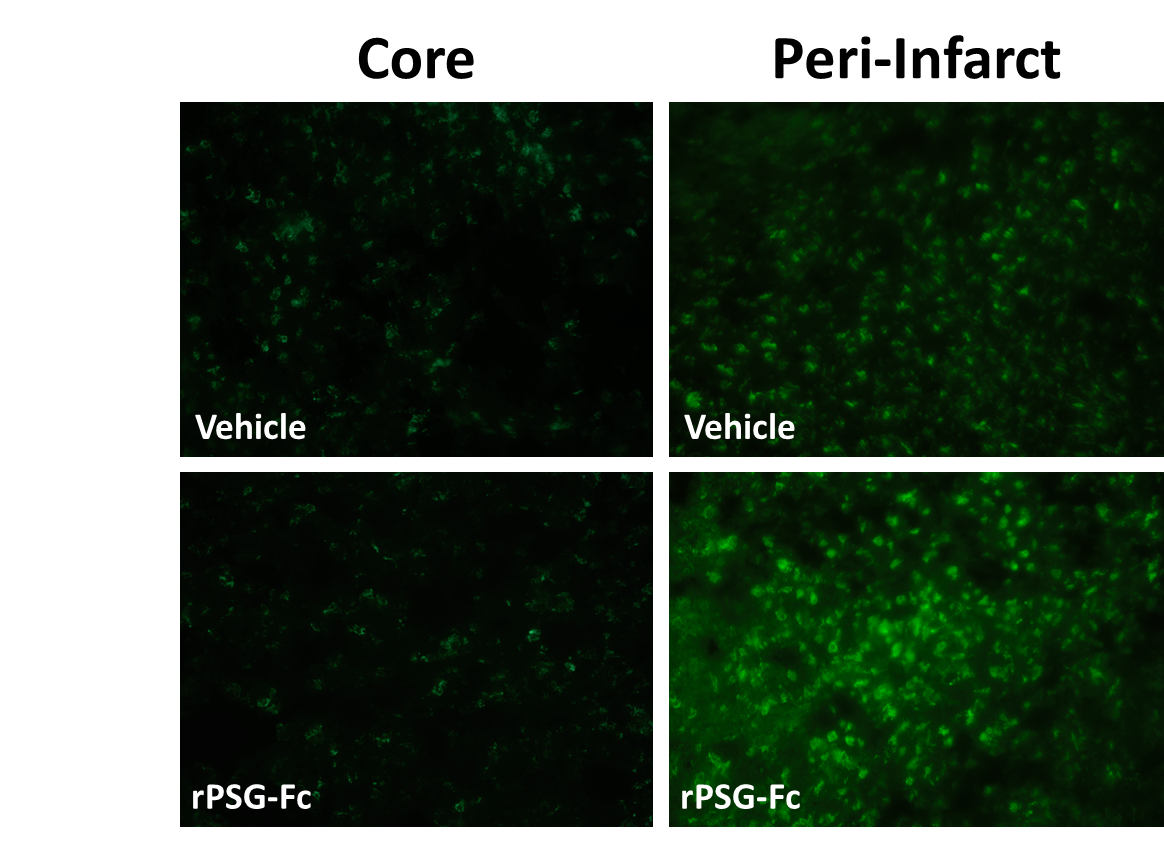


**Supplemental Figure 4:** Representative immunofluorescence images of Iba-1 staining in the core and peri-infarct zones of mice post-pMCAO (t = 5 days). Images acquired with the 20X objective lens of an Olympus BX51 microscope. Positive cells counted using ImageJ.
