# Supplementary material for: Recombinant pregnancy-specific glycoprotein-1-Fc reduces functional deficit in a mouse model of permanent brain ischaemia
Source: Brain Behav Immun Health. 2022 Aug 24;25:100497. doi: 10.1016/j.bbih.2022.100497 (PMC9475273; doi:10.1016/j.bbih.2022.100497)
Supplement: Supplementary Figure 6 [file mmc6.docx]

**Supplementary Figure 6**

**Article Title**

Recombinant pregnancy-specific glycoprotein-1-Fc reduces functional deficit in a mouse model of permanent brain ischaemia

**Journal**

Brain, Behaviour, and Immunity

**Authors**

Kyle Malone1,2, Jennifer A Shearer1,2, John M Williams3, Anne C Moore3, Tom Moore3*, Christian Waeber1,2*

**Affiliations**

^1^Department of Pharmacology and Therapeutics, Western Gateway Building, University College Cork, Cork, Ireland

^2^School of Pharmacy, University College Cork, Cork, Ireland

^3^School of Biochemistry and Cell Biology, University College Cork, Cork, Ireland.

**Corresponding Author Email**

Kyle.malone@ucc.ie


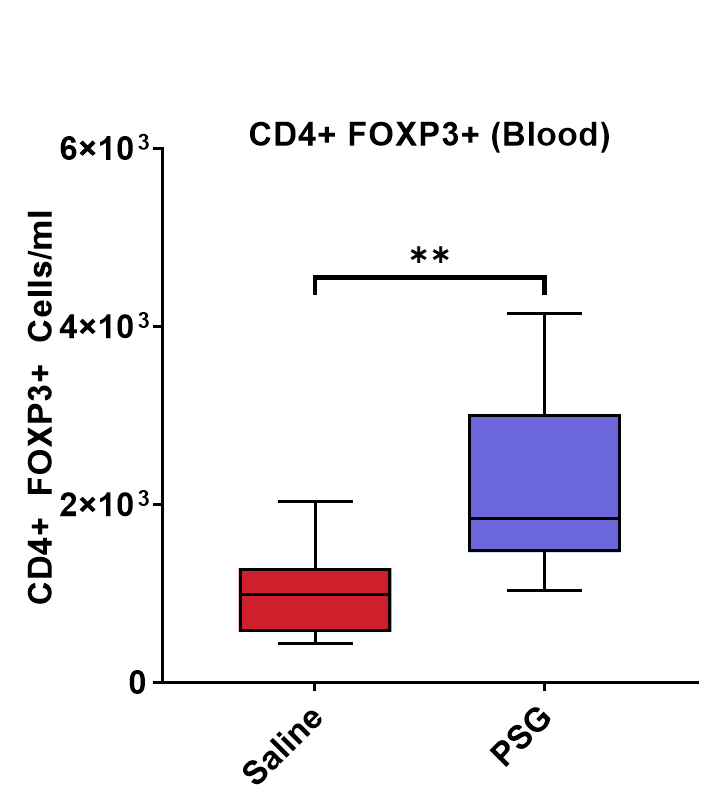


**Supplemental Figure 6:** Total CD4+ FoxP3+ cells in blood in response to either saline (n = 20) or rPSG1-Fc (n = 22) treatment post‑pMCAO (t =5 days). Two-sided, independent-samples T‑tests used to investigate differences between groups (* = *p*<0.05, ** = *p*<0.01, *** = *p*<0.001 as compared to saline). Box‑and-whisker plots exhibit 10-90 percentiles.

**
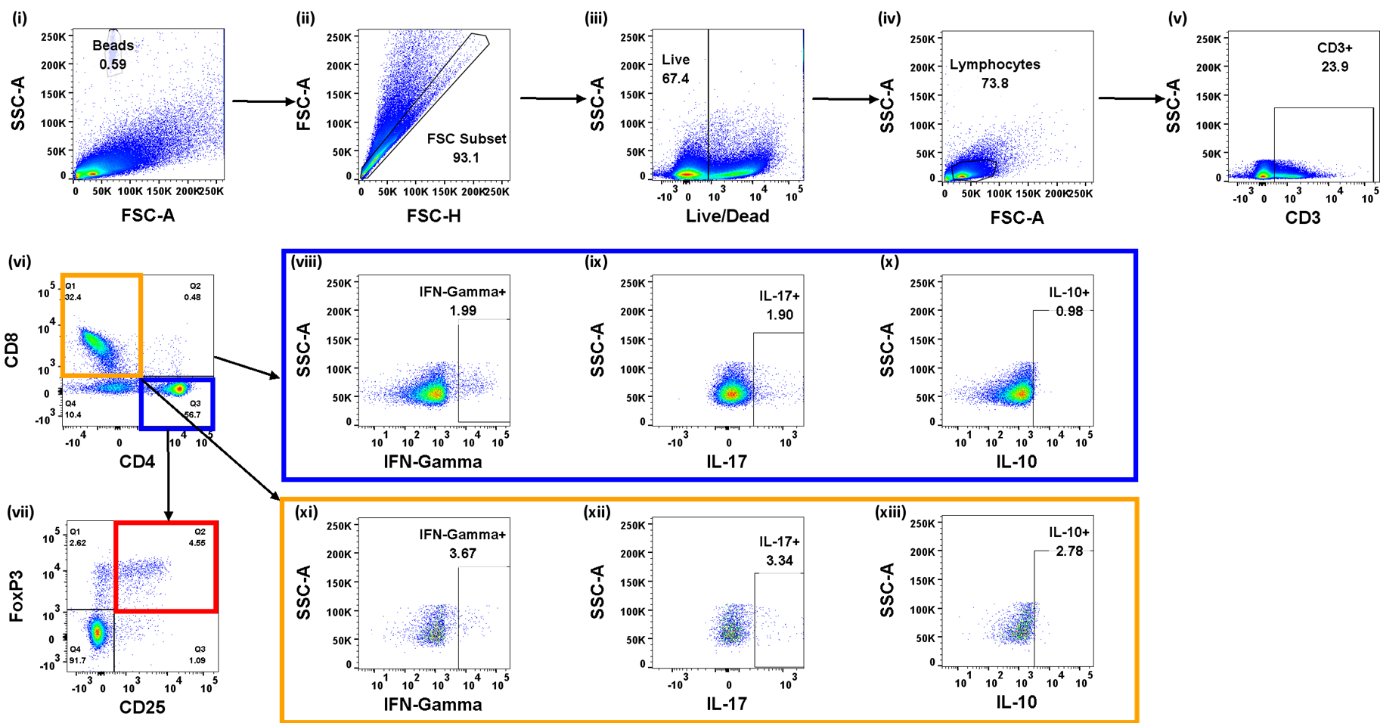
**

**Supplemental Figure 2:** Gating strategy for determination of Tregs (red frame), cytokine expression among CD4+ cells (blue frame), and cytokine expression among CD8+ cells (yellow frame) in representative mouse splenocytes 5 days post-brain ischaemia. (i) = Initial population, (ii) = singlets, (iii) = Live cells, (iv) = lymphocytes, (v) = CD3+ cells (T cells), (vi) = CD4+ vs. CD8+ cells, (vii) = CD4+ CD25+ FoxP3+ cells (quadrant in red) designated Tregs, (viii) = CD4+ IFNγ+ cells, (ix) = CD4+ IL‑17A+ cells, (x) = CD4+ IL-10+ cells, (xi) = CD8+ IFNγ+ cells, (xii) = CD8+ IL-17A+ cells, (xiii) = CD8+ IL-10+ cells. All gates were determined by both negative cells and fluorescence minus one controls.
